# Supplementary material for: The interactome of intact mitochondria by cross-linking mass spectrometry provides evidence for coexisting respiratory supercomplexes
Source: Mol Cell Proteomics. 2017 Dec 8;17(2):216–32. doi: 10.1074/mcp.RA117.000470 (PMC5795388; doi:10.1074/mcp.RA117.000470)
Supplement: Supplemental Data [file supp_RA117.000470_133922_0_supp_23978_fzffwf.zip › spectra_annotation/mito_DR_spectra_annotation/374-1-16-1-18-1.pdf]

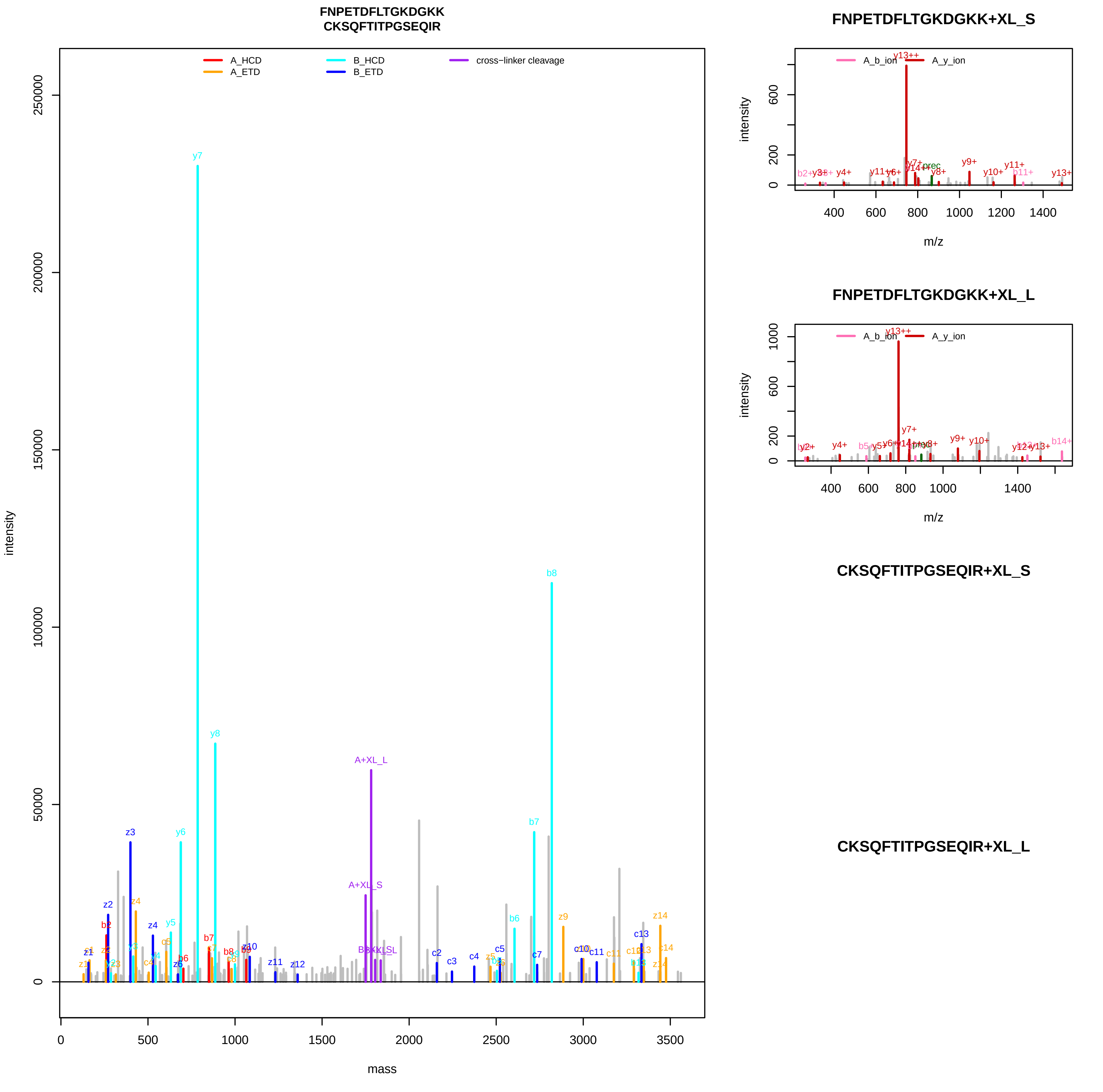
FNPETDFLTGKDGKK
CKSQFTITPGSEQIR
A_HCD
A_ETD
B_HCD
B_ETD
cross−linker cleavage
intensity
mass
0
50000
100000
150000
200000
250000
0
500
1000
1500
2000
2500
3000
3500
y7
b8
y8
A+XL_L
A+XL_S
z3
y6
b7
z2
b2
z4
y5
z9
z14
c13
b6
c5
c7
c10
c11
z11
z12
c2
c3
c4
FNPETDFLTGKDGKK+XL_S
A_b_ion
A_y_ion
y13++
m/z
400
600
800
1000
1200
1400
0
200
600
FNPETDFLTGKDGKK+XL_L
1000
CKSQFTITPGSEQIR+XL_S
CKSQFTITPGSEQIR+XL_L
